# Hyperforin potentiates polymyxin B against multidrug-resistant Gram-negative pathogens via membrane disruption, biofilm eradication, and oxidative stress

**DOI:** 10.1128/aac.01007-25

**Published:** 2025-11-12

**Authors:** Maytham Hussein, Simon Crawford, Mark Baker, Holly Floyd, Rafah Allobawi, Mark A. T. Blaskovich, Gauri G. Rao, Johannes Zuegg, Jian Li, Tony Velkov

**Affiliations:** 1 Department of Pharmacology, Monash Biomedicine Discovery Institute, Monash University 534222, Clayton, Victoria, Australia; 2 Ramaciotti Centre for Cryo-EM, Monash Biomedicine Discovery Institute, Monash University 214149, Clayton, Victoria, Australia; 3 Discipline of Biological Sciences, Priority Research Centre in Reproductive Biology, Faculty of Science and IT, University of Newcastle, University Drive 622829, Callaghan, New South Wales, Australia; 4 Institute of Molecular Biosciences, The University of Queensland 1974, Brisbane, Queensland, Australia; 5 Centre for Superbug Solutions and ARC Training Centre for Environmental and Agricultural Solutions to Antimicrobial Resistance, Institute for Molecular Bioscience, The University of Queensland 85088, Saint Lucia, Queensland, Australia; 6 Titus Family Department of Clinical Pharmacy, University of Southern California 5116, Los Angeles, California, USA; 7 Department of Microbiology, Monash Biomedicine Discovery Institute, Monash University 214149, Clayton, Victoria, Australia; University of Houston, Houston, Texas, USA

**Keywords:** polymyxin B, hyperforin, antimicrobial resistance, synergy, biofilm eradication

## Abstract

The escalating spread of multidrug-resistant (MDR) Gram-negative pathogens, particularly *Pseudomonas aeruginosa*, *Klebsiella pneumoniae*, and *Acinetobacter baumannii*, has severely undermined the efficacy of polymyxin B, one of the few remaining last-line antibiotics. Here, we identify hyperforin, a natural product derived from *Hypericum perforatum*, as a potent polymyxin B adjuvant capable of restoring its antibacterial activity against clinical Gram-negative isolates with high-level polymyxin resistance. Using a suite of *in vitro* assays, including fractional inhibitory concentration index, time-kill kinetics, biofilm eradication, membrane integrity assays, and ultrastructural imaging of the bacterial outer membrane, we show that hyperforin significantly enhances polymyxin B-mediated bacterial killing. Microscopy and *N*-phenyl-1-naphthylamine uptake are consistent with membrane perturbation, and increased intracellular reactive oxygen species is consistent with oxidative stress under combination treatment. Taken together, these orthogonal readouts support a working model of complementary membrane perturbation and oxidative stress. Remarkably, the observed synergistic effects occur at concentrations potentially achievable in the epithelial lining fluid of the lungs, providing a testable rationale for localized lung delivery (e.g., via a dry-powder inhaler). These findings unveil a promising therapeutic strategy that repurposes a bioactive phytochemical to potentiate polymyxins against otherwise untreatable polymyxin-resistant MDR Gram-negative infections.

## INTRODUCTION

Antimicrobial resistance poses a severe global health threat, compromising the efficacy of current antibiotics and leading to high mortality and morbidity rates (1, 2). Multidrug-resistant (MDR) Gram-negative bacteria, including *Pseudomonas aeruginosa*, *Klebsiella pneumoniae*, and *Acinetobacter baumannii*, are associated with life-threatening infections due to their capacity to resist conventional antibiotics through multiple mechanisms, such as efflux pump overexpression, enzymatic inactivation, and membrane remodeling (3). The resurgence of polymyxins, which serve as last-resort antibiotics for MDR infections, has provided a critical treatment option. However, the growing resistance to polymyxins has significantly limited their clinical effectiveness, necessitating alternative strategies to enhance their antibacterial activity (4–6).

Polymyxins, namely polymyxin B and colistin, are last-resort cationic lipopeptides used against Gram-negative pathogens that are resistant to almost all other antibiotics (7). They exert their antimicrobial effect primarily by disrupting the outer membrane (OM) of Gram-negative bacteria through direct interaction with the lipid A component of lipopolysaccharide (LPS), leading to membrane permeabilization and bacterial cell death (8). Despite their potent antibacterial effects, their clinical use is hindered by dose-dependent nephron- and neuro-toxicities (9). Additionally, monotherapy with polymyxins often fails to achieve complete bacterial eradication due to resistance mechanisms such as LPS modification and LPS loss from the OM (seen only in *A. baumannii*) (10). These limitations have driven interest in combination therapy to enhance efficacy and counteract resistance development.

St. John’s Wort (*Hypericum perforatum*), traditionally used for wound healing, burns, and ulcers, is now primarily known for treating anxiety and depression (11, 12). Hyperforin has been demonstrated to display potent antibacterial activity, inhibiting the growth of the Gram-positive pathogen *Staphylococcus aureus*, with minimum inhibitory concentration (MIC) values as low as 0.1 µg/mL (13–16). Furthermore, one study reported greater anti-staphylococcal activity against methicillin-resistant *S. aureus* compared to methicillin-sensitive *S. aureus* (17). In contrast, hyperforin’s activity against Gram-negative bacteria has been poorly characterized, with available studies reporting significantly higher MIC values (>100 µg/mL) against *Escherichia coli* and *P. aeruginosa* (15). In view of hyperforin’s promising efficacy against resistant Gram-positive pathogens and historical use in treating skin infections, further studies are warranted to assess its potential against MDR Gram-negative bacteria.

Despite its limited standalone activity against Gram-negative bacteria, hyperforin has not been investigated for its synergistic potential to enhance the efficacy of last-line antibiotics. In this study, we evaluated the synergistic potential of hyperforin in combination with polymyxin B against a panel of polymyxin-susceptible and polymyxin-resistant clinical isolates of *P. aeruginosa*, *K. pneumoniae*, and *A. baumannii*. Through a comprehensive set of *in vitro* assays, including fractional inhibitory concentration index (FICI), time-kill kinetics, biofilm eradication, intracellular reactive oxygen species (ROS) measurement, and ultrastructural analysis using both scanning and transmission electron microscopy, we found that hyperforin markedly potentiated the bactericidal activity of polymyxin B at clinically relevant concentrations. These findings suggest that combining hyperforin with polymyxin B may represent a promising therapeutic strategy to overcome resistance and improve clinical outcomes in MDR Gram-negative lung infections.

## MATERIALS AND METHODS

### Chemicals and reagents

Polymyxin B (Beta Pharma, China; Batch no. 20120204) was freshly dissolved in Milli-Q water and sterilized using a 0.22 µm syringe filter (Sartorius, Australia). Hyperforin (Henan Chemical Co., China; CAS no. 11079-53-1) was dissolved in methanol (MeOH). Cation-adjusted Mueller-Hinton broth (CAMHB) medium (Oxoid, England) was prepared from Mueller-Hinton broth (MHB) adjusted with 22.5 mg/L CaCl_2_ and 11.25 mg/L MgCl_2_. All other reagents were obtained from Sigma-Aldrich (Australia) and were of the highest commercially available grade.

### Bacterial isolates

This study included 32 clinical isolates comprising *K. pneumoniae* (*n* = 10), *P. aeruginosa* (*n* = 11), and *A. baumannii* (*n* = 11). Strains include both polymyxin B-susceptible and polymyxin B-resistant phenotypes. Detailed strain information and susceptibility profiles are provided in Table 1. The antibiogram for the MDR *P. aeruginosa* strain FADDI-PA070 is provided in Table S1.

**TABLE 1:** Antimicrobial activity of polymyxin B (PMB) and hyperforin (HF) in monotherapy and in combination

Strain^*a*^	MIC (μg/mL)		FIC^*b*^		
	PMB	HF	PMB	HF	PMB/HF
*K. pneumoniae*					
ATCC 13883	0.25	>128	0.06	0.5	0.25
FADDI-KP009	1	>128	0.25	0.12	0.25
FADDI-KP010	0.5	128	0.06	0.12	0.12
FADDI-KP030	32	>128	8.0	4.0	0.26
ATCC 13883R	>128	>128	2.0	2.0	0.01
FADDI-KP012	16	>128	1.0	2.0	0.07
Kp BM1	0.5	64	0.12	2.0	0.28
FADDI-KP005	1	128	0.12	1.0	0.13
FADDI-KP003	32	>128	0.5	1.0	0.01
FADDI-KP006	1	>128	0.12	1.0	0.12
*P. aeruginosa*					
PAO1	1	>128	1.0	256.0	2.0
FADDI-PA064 n/m	128	4	2.0	1.0	0.26
FADDI-PA065 m	128	>128	0.25	0.125	0.002
FADDI-PA007 n/m	0.5	>128	0.12	0.125	0.25
FADDI-PA006 m	8	>128	0.25	0.12	0.03
PA14	1	>128	0.25	0.12	0.25
FADDI-PA091	1	>128	1.0	256	2.0
FADDI-PA092	16	>128	2.0	1.0	0.12
FADDI-PA093	32	>128	16	1.0	0.50
FADDI-PA070 n/m	128	>128	2.0	0.5	0.01
FADDI-PA067 n/m	32	>128	2.0	1.0	0.06
*A. baumannii*					
ATCC 19606	0.5	>128	0.25	1.0	0.50
FADDI-AB065	64	0.25	64	0.25	2.0
ATCC 17978	0.5	>128	0.12	0.5	0.25
FADDI-AB225	32	>128	4.0	2.0	0.13
FADDI-AB060	64	>128	2.0	1.0	0.03
FADDI-AB146	16	>128	2.0	0.12	0.12
FADDI-AB145	4	>128	2.0	0.12	0.50
FADDI-AB151	4	>128	0.25	1.0	0.06
FADDI-AB143	16	>128	2.0	1.0	0.12
FADDI-AB144	8	>128	2.0	1.0	0.25

^*a*^ n/m, nonmucoid; m, mucoid.

^*b*^ FIC = FIC index = (MIC polymyxin B in combination with hyperforin/MIC polymyxin B monotherapy) + (MIC hyperforin in combination with polymyxin B/MIC hyperforin monotherapy); synergy FIC < 0.5; additivity FIC = 0.5–1.0; indifference FIC = 1–4; antagonism FIC ≥ 4 (not observed).

### Determination of MICs and FICI

MICs of polymyxin B and hyperforin were determined using the broth microdilution method following CLSI guidelines (18). Hyperforin was dissolved in MeOH and diluted in CAMHB broth. Polymyxin B was freshly prepared in Milli-Q water and sterilized using a 0.22  µm syringe filter (Sartorius). Bacterial suspensions were adjusted to approximately 0.5 McFarland standard. For each well, 100  µL of the bacterial suspension was mixed with 100  µL of serially diluted antibiotic solution, resulting in a final volume of 200  µL per well. Plates were incubated at 37°C for 18–20 h. The MIC was defined as the lowest concentration of drug that completely inhibited visible growth (18).

Polymyxin B MICs were interpreted using a combination of EUCAST and CLSI breakpoints. For *K. pneumoniae*, the EUCAST 2025 epidemiological cut-off value (ECOFF) of ≤2  mg/L was applied to define wild-type susceptibility (19). For *P. aeruginosa* and *A. baumannii*, CLSI M100-ED34:2024 clinical breakpoints were used: susceptible ≤2  µg/mL, intermediate = 4  µg/mL, and resistant ≥8 µg/mL (20).

Checkerboard assays were employed to assess synergy between polymyxin B and hyperforin. FICI was calculated as follows:

FICI = (MIC of drug A in combination ÷ MIC of drug A alone) + (MIC of drug B in combination ÷ MIC of drug B alone).

FICI values were interpreted as follows: synergism FIC ≤ 0.5; addition FIC = 0.5–1.0; indifference FIC = 1–4; antagonism FIC ≥ 4 (21). An MIC of 128 µg/mL for hyperforin was used to calculate the FICI scores. Hyperforin concentrations required to enhance polymyxin B activity had no measurable inhibitory effect when used alone.

### Static time-kill assays

Static time-kill assays were conducted to evaluate the bactericidal activity of polymyxin B, hyperforin, and their combination against selected Gram-negative clinical isolates. Overnight cultures of *P. aeruginosa*, *K. pneumoniae*, and *A. baumannii* strains were prepared by inoculating single colonies into 10  mL of CAMHB and incubating at 37°C with shaking at 150  rpm for 16  h. The cultures were then diluted into fresh CAMHB and incubated for 2 h to reach early log phase (OD_600_ ~ 0.5).

For each assay, early log-phase cultures were diluted 1:100 into fresh CAMHB in 50  mL glass Erlenmeyer tubes to achieve a final inoculum of ~1 × 10^6^ CFU/mL. The cultures were then treated with polymyxin B (0.125–4 mg/L), hyperforin (4  mg/L), or their combination. A vehicle control tube containing 0.08% MeOH, matching the final solvent concentration used for hyperforin, was included to account for any solvent effects. Untreated control groups received no drug or solvent. All tubes were incubated at 37 °C with shaking at 150 rpm. Samples were collected at 0, 1, 4, 8, and 24 h post-treatment, serially diluted in sterile saline, and plated for viable colony counts (CFU/mL) using an automatic spiral plater (Don Whitley Scientific, Australia). Colonies were enumerated after overnight incubation on nutrient agar plates at 37°C.

Bactericidal activity was defined as a reduction of ≥3-log₁₀ CFU/mL from the initial inoculum at any time point, while synergy was defined as a ≥2-log₁₀ CFU/mL reduction achieved by the combination compared to the most active single agent (6, 22).

### Minimum biofilm eradication concentration (MBEC) assay

The MBEC assay was performed, with minor modifications to a previously described method (23), to evaluate the biofilm eradication potential of polymyxin B, hyperforin, and their combination against *P. aeruginosa* FADDI-PA067, a cystic fibrosis isolate (polymyxin B MIC = 32  mg/L). Biofilms were established in flat-bottomed 96-well microtiter plates (Corning, Cat. no. 3370). A bacterial suspension was adjusted to approximately 0.5 McFarland standard using 0.9% saline. Each well received 100  µL of bacterial suspension and 100  µL of fresh CAMHB (no drug).

Plates were then incubated at 37°C for 48  h in a humidified chamber to allow for mature biofilm formation while minimizing evaporation and edge effects. After incubation, planktonic cells were removed by inverting the plates over a disinfectant reservoir, followed by three gentle washes with phosphate-buffered saline (PBS). Serial dilutions of polymyxin B (0.0625–128  mg/L), either alone or in combination with a fixed concentration of hyperforin (4 mg/L), were then prepared in fresh CAMHB and added to the established biofilms (200 µL per well). Plates were further incubated at 3°C for 24  h.

Following treatment, wells were washed three times with PBS and stained with 220 µL of 0.1% crystal violet (Sigma-Aldrich) for 10  min at room temperature. Excess stain was removed, and the wells were washed thrice with PBS. Plates were air-dried before adding 220  µL of 30% (v/v) acetic acid (Sigma-Aldrich) to solubilize the retained crystal violet. After 10 min, 200 µL of the solubilized solution was transferred to a new 96-well plate, and absorbance was measured at 590 nm using a Tecan Infinite M200 microplate reader (Tecan Group Ltd, Männedorf, Switzerland).

The percentage of biofilm eradication was calculated relative to the untreated control using the following formula:


Eradication (%) = (1 − (Treated OD / Control OD)) × 100

MBEC was defined as the lowest concentration of polymyxin B (alone or in combination) that achieved ≥70% reduction in biofilm biomass relative to the untreated control, consistent with biomass-based eradication thresholds reported in the literature (24, 25). Statistical analyses of MBEC data were analyzed by two-way ANOVA (factors: treatment and concentration; interaction included) using GraphPad Prism v10. Tukey’s post hoc tests compared treatments within each concentration, reporting multiplicity-adjusted *P* values (*α* = 0.05).

### Scanning and transmission electron microscopy (SEM/TEM)

SEM and TEM were performed as previously described (26), using *P. aeruginosa* FADDI-PA070 treated with polymyxin B (8 mg/L), hyperforin (4 mg/L), or their combination for 1 and 4  h.

### OM permeability assay

OM disruption was evaluated using the *N*-phenyl-1-naphthylamine (NPN) uptake assay in *P. aeruginosa* FADDI-PA070 (polymyxin B MIC = 128  mg/L), following a previously described method with minor modifications (27). Bacterial cells were grown overnight at 37°C in MHB, harvested by centrifugation, washed, and resuspended in 5  mM HEPES buffer (pH 7.2) supplemented with 20  mM glucose to an OD_600_ of approximately 0.5. NPN (Sigma-Aldrich) was added to a final concentration of 10  µM. Cells were treated with polymyxin B (8 mg/L), hyperforin (4 mg/L), or their combination in black 96-well microplates. Fluorescence was measured at excitation 350 nm and emission 420 nm using a BMG Labtech CLARIOstar plate reader with constant instrument gain. Readings were collected at 0, 5, 10, 15, and 20 min, and the peak fluorescence at 20 min was used for comparative analysis.

### Intracellular ROS assay

Intracellular ROS production was measured using 2′,7′-dichlorodihydrofluorescein diacetate (DCFH-DA; Sigma-Aldrich) as previously described with minor modifications (28). *P. aeruginosa* FADDI-PA070 (polymyxin B MIC = 128 mg/L) was grown overnight in CAMHB and incubated for ~2 h to mid-log phase (OD_600_ ~ 0.2–0.3). Cells were then washed and resuspended in PBS for dye loading. Bacterial suspensions were incubated with 10 µM DCFH-DA at 37°C for 30 min in the dark to allow dye uptake and intracellular conversion.

After loading, cells were washed twice with PBS and treated with polymyxin B (8 mg/L), hyperforin (4 mg/mL), or their combination in black 96-well plates (Corning, USA). A vehicle control (0.08% MeOH in PBS) and untreated control (PBS only) were included. Fluorescence intensity was recorded after 30 min of incubation at 37°C using a CLARIOstar plate reader (BMG LABTECH) at excitation 488 nm and emission 525 nm. Data were normalized to the untreated control and presented as relative fluorescence units (RFU). Each condition was tested in triplicate and repeated independently twice.

### Cytotoxicity assay

HEK-293 cells (ATCC CRL-1573) were cultured in Dulbecco’s modified Eagle medium (DMEM; Gibco, Thermo Fisher Scientific) supplemented with 10% fetal bovine serum (FBS; Gibco, Thermo Fisher Scientific). Cells were counted with a Neubauer hemocytometer and seeded into 384-well, tissue-culture-treated plates (Corning) already containing test compounds to a density of 5,000 cells/well in a final volume of 50 µL. Plates were incubated for 20 h at 37°C, 5% CO_2_.

Cell viability was measured by resazurin reduction: 5 µL of 25 µg/mL resazurin (Sigma-Aldrich/Merck) was added to each well (final 2.3 µg/mL), followed by 3 h incubation before fluorescence reading (Ex 560/10 nm; Em 590/10 nm) on a Tecan Infinite M1000 Pro plate reader (Tecan Group Ltd), using the instrument’s Optimal (automatic) PMT gain setting to maximize dynamic range. CC_50_ values were obtained by four-parameter logistic fits of percent inhibition versus log_10_(concentration). Technical replicates were averaged prior to fitting. Each concentration was tested in ≥3 technical replicates per experiment, and results reflect two independent experiments.

### Hemolysis assay

Human whole blood was obtained from Australian Red Cross LifeBlood under UQ HREC approval 2020001239. Whole blood was washed 3× with 0.9% NaCl (normal saline; Sigma-Aldrich/Merck) (three volumes per wash) and resuspended in saline to 0.5 × 10^8^ cells/mL, as determined by manual counting with a Neubauer hemocytometer. Washed cells were dispensed into 384-well polystyrene plates (Corning) pre-loaded with test compounds to a final volume of 50 µL per well, shaken 10 min, and incubated 1 h at 37°C. Plates were then centrifuged at 1,000 × *g* for 10 min, and 25 µL of supernatant was transferred to a fresh 384-well polystyrene plate (Corning). Hemoglobin release was quantified by measuring A405 on a Tecan Infinite M1000 Pro monochromator plate reader (Tecan Group Ltd). Percent hemolysis was calculated relative to vehicle (0%) and a complete-lysis control (melittin; Sigma-Aldrich/Merck). HC_10_ and HC_50_ (µg/mL) were obtained by four-parameter logistic fits of % hemolysis versus log_10_(concentration). Assays were performed with *n* = 3 independent donors (biological replicates), each with ≥3 technical replicates per concentration.

## RESULTS

### Synergistic effects of polymyxin B and hyperforin determined by FICI

The synergistic antibacterial activity of polymyxin B and hyperforin as monotherapies and in combination was evaluated against a panel of clinical isolates of *K. pneumoniae*, *P. aeruginosa*, and *A. baumannii* (Table 1). The combinations demonstrated synergistic antibacterial activity, assessed by the FICI, where synergy was defined as FICI < 0.5, additivity as FICI between 0.5 and 1.0, indifference as FICI between 1 and 4, and antagonism as FICI ≥ 4 (21, 29).

Against *K. pneumoniae*, synergy was observed across all tested strains, including polymyxin-susceptible isolates (e.g., ATCC 13883 [FICI = 0.015] and FADDI-KP009 [FICI = 0.25]) and polymyxin-resistant isolates (e.g., FADDI-KP030 [FICI = 0.26] and ATCC 13883R [FICI = 0.01]). Similarly, in *P. aeruginosa*, synergistic interactions were observed for most strains, including both polymyxin-susceptible and polymyxin-resistant isolates. Notable examples include FADDI-PA007 (FICI = 0.025), PA14 (FICI = 0.25), FADDI-PA065 (FICI = 0.002), and FADDI-PA006 (FICI = 0.03). However, an additive effect was noticed with one polymyxin-resistant strain, FADDI-PA093 (polymyxin B MIC = 32 mg/L; FICI = 0.5). Indifference (FICI = 2.0) was observed against two polymyxin-susceptible strains: PAO1 (polymyxin B MIC = 1 mg/L) and FADDI-PA091 (polymyxin B MIC = 1 mg/L).

In *A. baumannii*, a similar pattern to *P. aeruginosa* was observed, albeit with a more pronounced synergistic effect. Strong synergy was recorded across various strains, including both polymyxin-susceptible and polymyxin-resistant isolates, with FICI values ranging from 0.06 to 0.25. Additive effects were observed in two strains: ATCC 19606 (FICI = 0.5) and FADDI-AB145 (FICI = 0.5). One strain, FADDI-AB065 (polymyxin B MIC = 64 mg/L; FICI = 2.0), showed an indifferent effect. Notably, this strain is LPS-deficient, lacking an OM LPS, which might support the hypothesis that polymyxin B permeabilizes the OM of Gram-negative bacteria and enables hyperforin to access its intracellular target, namely the peptidoglycan layer or other potential intracellular sites (6, 30). Interestingly, hyperforin exhibited a notably low MIC of 0.25 mg/L against FADDI-AB065, suggesting that the absence of LPS may enhance its access or antibacterial activity.

### Static time-kill assays of polymyxin B-hyperforin combinations against selected strains

**Fig 1:**
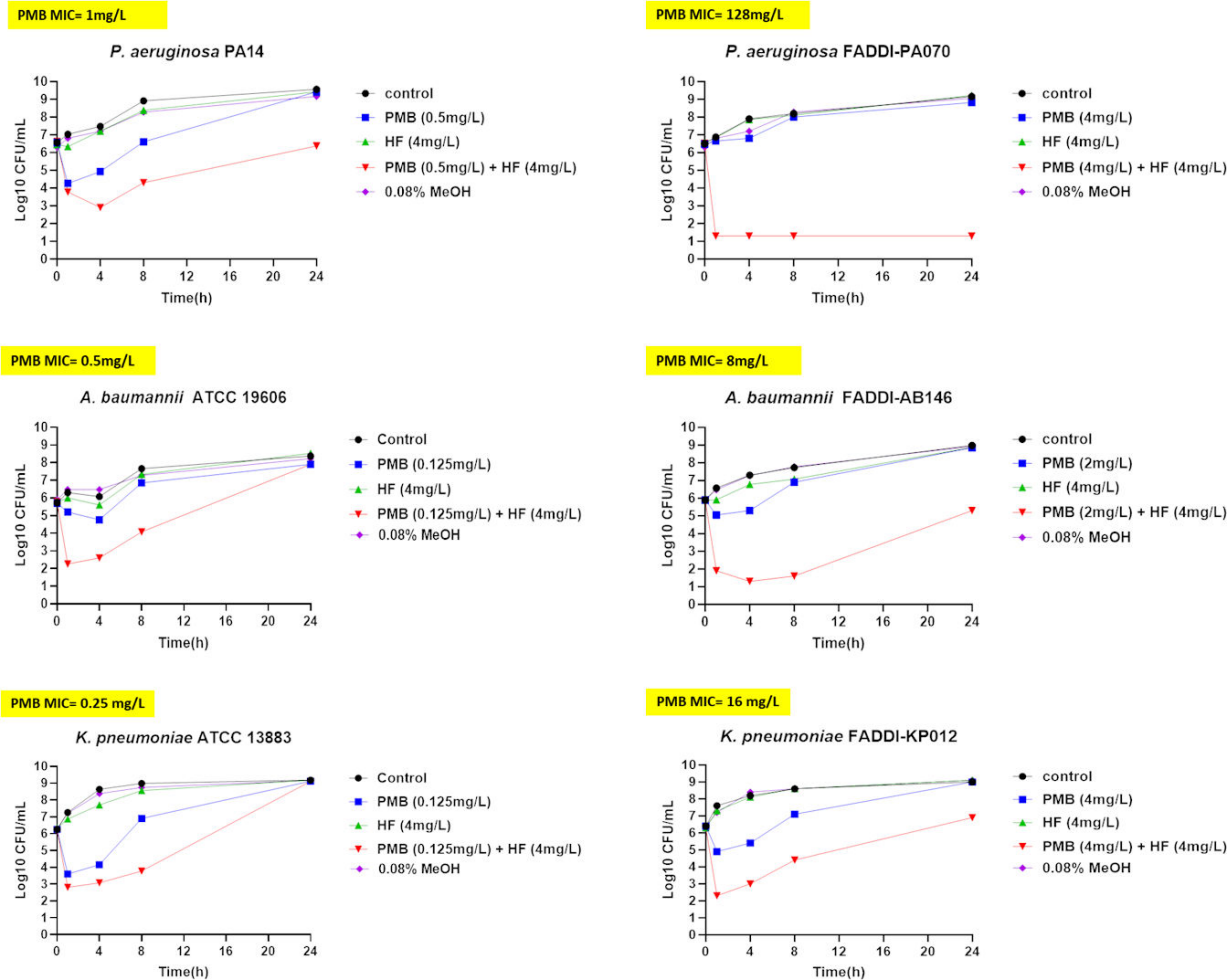
Time-kill kinetics of polymyxin B (PMB), hyperforin (HF), and their combination against *P. aeruginosa*, *A. baumannii*, and *K. pneumoniae* strains. PMB-susceptible strains: *P. aeruginosa* PA14 (MIC 1 mg/L), *A. baumannii* ATCC 19606 (MIC 0.5 mg/L), *K. pneumoniae* ATCC 13883 (MIC 0.25 mg/L). PMB-resistant strains: *P. aeruginosa* FADDI-PA070 (128 mg/L), *A. baumannii* FADDI-AB146 (8 mg/L), *K. pneumoniae* FADDI-KP012 (16 mg/L). HF MICs were >128  mg/L for all strains. HF fixed at 4 mg/L; PMB tested at a single sub-MIC concentration per isolate, and the same PMB level was used in PMB-alone and PMB + HF arms. Data are presented as means of three independent cultures. Vertical bars represent standard deviations; error bars are too small to be visible in the graphs.

Static time-kill assays were performed to evaluate the bactericidal activity of polymyxin B, hyperforin, and their combination against four clinical strains each of *P. aeruginosa*, *A. baumannii*, and *K. pneumoniae* (Fig. 1; Fig. S1). Two representative strains from each species are shown in Fig. 1, while the remaining six are included in Fig. S1. Bactericidal activity was defined as a reduction of ≥3-log₁₀ CFU/mL from the initial inoculum at any time point, while synergy was defined as a ≥2-log₁₀ CFU/mL reduction achieved by the combination compared to the most active single agent (6, 22).

The combination therapy induced rapid killing against *P. aeruginosa* PA14 (polymyxin B MIC = 1 mg/L), achieving a >4.5-log_10_ decrease in bacterial counts at 8 h, which surpassed the effect of polymyxin B monotherapy (2.3-log₁₀ reduction). At 24 h, regrowth was evident in the polymyxin B group, with only a 0.1-log_10_ reduction remaining, whereas the combination sustained a >3-log_10_ decrease. Against the highly resistant *P. aeruginosa* FADDI-PA070 (polymyxin B MIC = 128 mg/L), polymyxin B alone exhibited minimal activity, with bacterial reductions ranging from 0.2 to 1.1 log_10_ units across all timepoints. In contrast, the combination demonstrated strong and sustained killing, reaching a >7-log_10_ reduction at 24 h.

A nearly similar pattern was observed against *A. baumannii* strains. The combination induced a rapid 4-log_10_ decrease in *A. baumannii* ATCC 19606 (polymyxin B MIC = 1 mg/L) as early as 1 h, clearly surpassing the activity of polymyxin B monotherapy, which achieved only a 1.1-log_10_ reduction. At 24 h, polymyxin B lost activity entirely, while the combination still maintained a minimal effect. Against the resistant strain FADDI-AB146 (polymyxin B MIC = 16 mg/L), polymyxin B alone produced no more than a 2-log_10_ decrease at any timepoint. In contrast, the combination resulted in a 6-log_10_ reduction at 8 h and retained greater activity at 24 h.

A comparable trend was noted in the time-kill assays of *K. pneumoniae* strains. In ATCC 13883 (polymyxin B MIC = 0.25 mg/L), the combination achieved a 5.6-log_10_ reduction at 4 h, outperforming polymyxin B, which reduced CFU by 4.5 log_10_. Regrowth was observed in both groups at 24 h, although the combination maintained slightly greater activity. In the resistant strain FADDI-KP012 (polymyxin B MIC = 16 mg/L), polymyxin B alone achieved 2.7-log_10_ and 1.5-log_10_ reductions at 1 and 8 h, respectively, while the combination resulted in a 5.2-log_10_ reduction at 4 h and remained more effective overall, despite partial regrowth at 24 h (2.1-log_10_ reduction).

The additional six strains presented in Fig. S1 followed consistent trends. In all cases, the combination provided improved bacterial killing over monotherapies, with the greatest benefit observed in polymyxin-resistant isolates, where monotherapy was largely ineffective. Hyperforin monotherapy showed no bacterial killing against any of the strains tested. Altogether, these findings demonstrate that the combination of polymyxin B and hyperforin substantially enhances bacterial killing against both susceptible and highly resistant Gram-negative pathogens.

### MBEC assay

**Fig 2:**
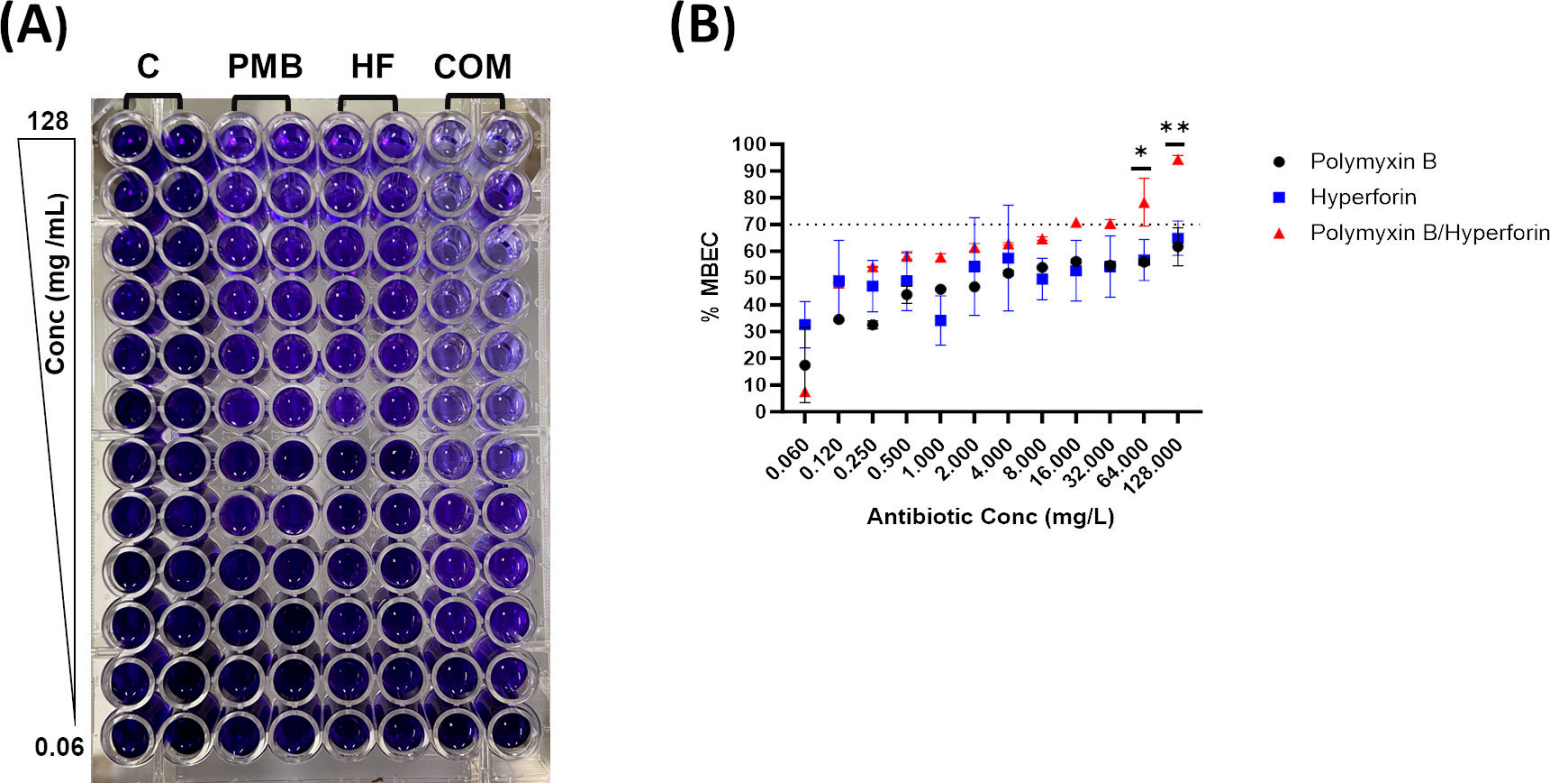
(**A**) Representative image of crystal violet-stained 96-well plate showing biofilm eradication in *P. aeruginosa* FADDI-PA067 following treatment with polymyxin B (0.0625–128 µg/mL), hyperforin (4  mg/L), and their combination. (**B**) Quantitative analysis of biofilm eradication based on crystal violet absorbance readings. MBEC values were calculated as percent reduction in biomass relative to the untreated control (cf. Materials and Methods). Data are presented as mean ± standard deviation (SD) from two independent replicates. Statistics: two-way ANOVA with Tukey’s multiple comparisons, comparing treatments within each concentration; symbols shown only where the combination differed from both monotherapies (conservative common level): *P* < 0.05 (*), *P* < 0.01 (**). The dotted line marks the 70% MBEC threshold.

To evaluate antibiofilm efficacy, a crystal violet staining assay was performed against a heavy biofilm forming cystic fibrosis isolate, *P. aeruginosa* FADDI-PA067 (PMB MIC = 32 mg/L; Fig. 2A and B). The biofilm eradication activity of polymyxin B, hyperforin (4 mg/L), and their combination was assessed across a polymyxin B concentration gradient (0.06–128 mg/L), with MBEC values reported as percent biomass remaining relative to the untreated control.

Polymyxin B and hyperforin monotherapies displayed limited antibiofilm activity, achieving ~60% eradication only at the highest tested concentration (128  µg/mL), indicating poor potency at clinically relevant doses (Fig. 2B). In contrast, the combination exceeded the prespecified 70% eradication threshold at 16 mg/L polymyxin B. Significant pairwise differences versus both monotherapies were first observed at ≥64 mg/L (Tukey, adjusted *P* < 0.05) and were sustained at higher concentrations (Fig. 2B). These concentrations fall within intrapulmonary exposures reported after aerosolized polymyxin B (ELF ≈ 20.6–97.6 mg/L) (31, 32). Crystal violet-stained microplates visually confirmed these findings, with visibly reduced biomass in combination-treated wells (Fig. 2A). Collectively, these results highlight polymyxin B-hyperforin as a promising combination approach for biofilm-associated *P. aeruginosa* infections.

### SEM/TEM

**Fig 3:**
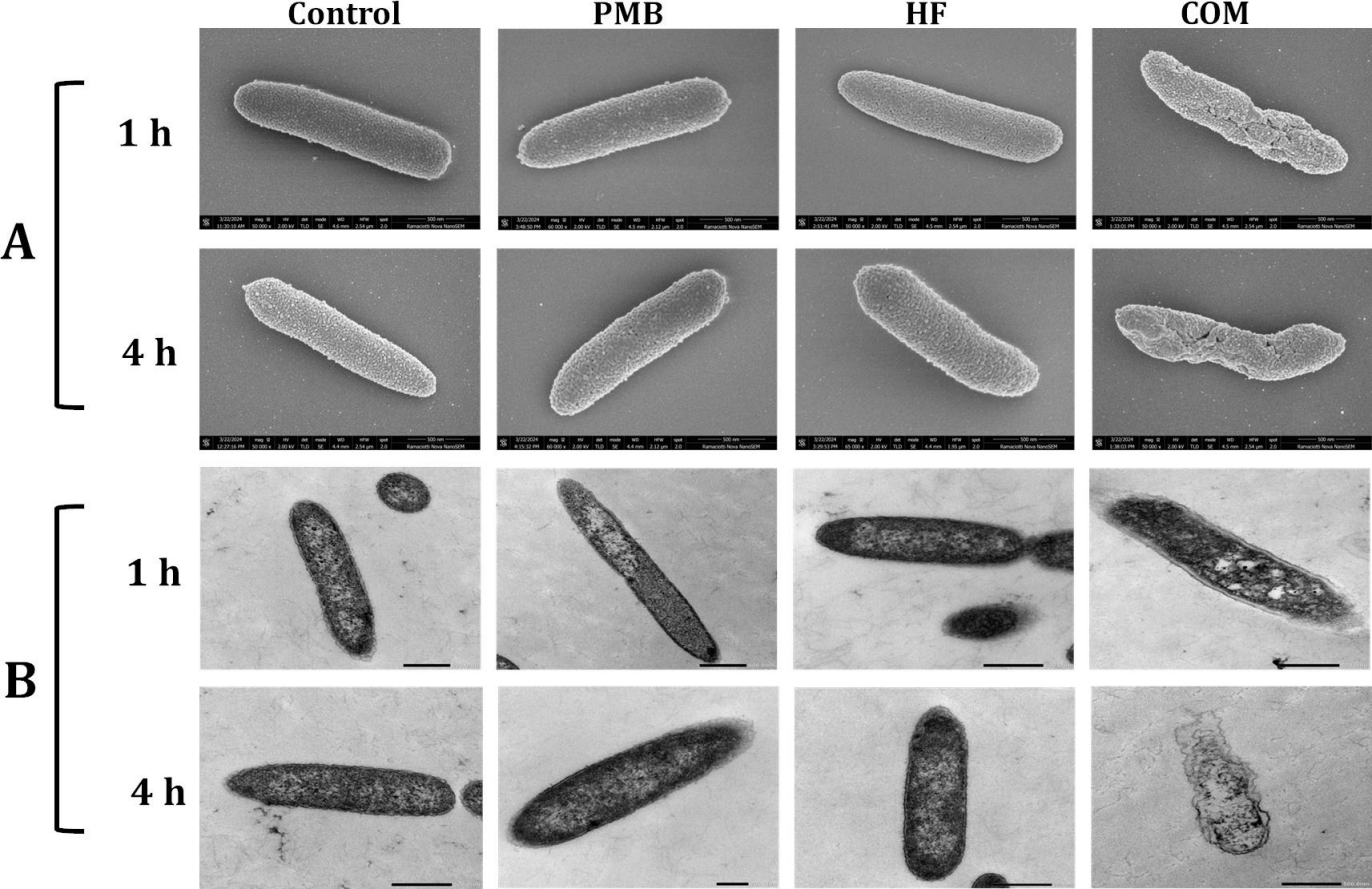
(**A**) Scanning (SEM) and (**B**) transmission (TEM) electron micrographs of *P. aeruginosa* FADDI-PA070 following treatment with polymyxin B (PMB, 8 mg/L), hyperforin (HF, 4 mg/L), or their combination (COM) at 1 and 4  h. SEM images are shown in the top two rows; TEM images in the bottom two rows. Scale bars, 500 nm.

To assess the morphological and ultrastructural impact of the polymyxin B and hyperforin combination on the OM of polymyxin-resistant *P. aeruginosa* FADDI-PA070 (PMB MIC = 128 mg/L), SEM and TEM were conducted at 1 and 4 h post-treatment (Fig. 3A and B). SEM imaging showed distinct morphological changes at both 1 and 4  h following combination treatment. At 1  h, cells exhibited early signs of surface wrinkling and envelope deformation. These alterations became markedly more severe at 4  h, with evident membrane corrugation, shrinkage, and collapse. In contrast, cells in the control and monotherapy groups maintained a smooth, intact rod-like morphology at both time points, underscoring the progressive membrane-disruptive effect of the combination therapy (Fig. 3A).

TEM imaging further confirmed significant structural damage after the combination treatment. At 1  h, cells showed early signs of membrane disruption, cytoplasmic granulation, and formation of electron-lucent vacuoles suggestive of cytoplasmic leakage. At 4  h, the damage was even more extensive, characterized by total loss of intracellular architecture and ghost-like appearances, indicating irreversible membrane failure and lysis. These effects were substantially more pronounced than those observed with monotherapies, supporting a synergistic mechanism involving severe membrane and cytoplasmic disruption (Fig. 3B).

These findings support the hypothesis that the combination of polymyxin B and hyperforin synergistically compromises bacterial membrane integrity, leading to rapid and extensive cellular disintegration in a highly resistant *P. aeruginosa* strain.

### OM permeabilization assay

**Fig 4:**
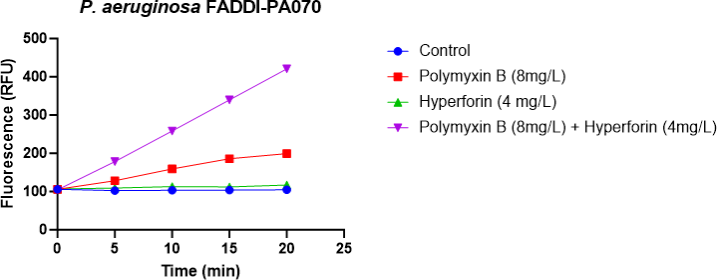
Outer membrane permeabilization of *P. aeruginosa* FADDI-PA070 assessed by NPN uptake assay. Bacterial cells (OD_600_ ~ 0.5) were treated with polymyxin B (8 mg/L), hyperforin (4 mg/L), or their combination. *N*-phenyl-1-naphthylamine (NPN, 10 µM) fluorescence was measured over 20 min using a CLARIOstar plate reader (excitation 350 nm, emission 420 nm), with constant instrument gain. Each condition was tested in triplicate. Data are presented as mean ± standard deviation (SD); error bars are not visible due to minimal variation between replicates.

To assess OM integrity, NPN uptake was measured in *P. aeruginosa* FADDI-PA070 (PMB MIC = 128 mg/L) following treatment with polymyxin B (8 mg/L), hyperforin (4 mg/L), or their combination (Fig. 4). The control and hyperforin-only groups exhibited minimal fluorescence over 20 min, indicating intact OMs. Polymyxin B alone caused a moderate, time-dependent increase in NPN fluorescence, reflecting partial permeabilization. In contrast, the combination of polymyxin B and hyperforin induced a marked and progressive increase in fluorescence, exceeding 400 RFU at 20 min. These findings demonstrate that hyperforin enhances polymyxin B-induced OM disruption, consistent with a synergistic effect on membrane permeabilization against this polymyxin-resistant strain.

### Intracellular ROS assay

**Fig 5:**
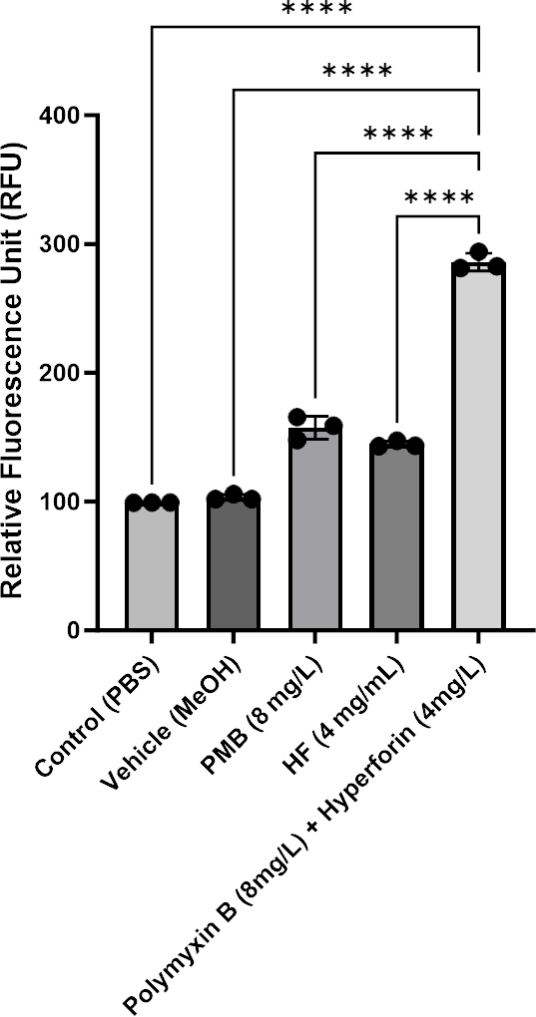
Intracellular reactive oxygen species (ROS) levels in *Pseudomonas aeruginosa* FADDI-PA070 following treatment with polymyxin B (8 mg/L), hyperforin (4 mg/mL), their combination, or vehicle control (0.08% MeOH). Fluorescence intensity was measured using a microplate reader (excitation 488 nm, emission 525 nm) and normalized to the untreated control (PBS). Data are presented as mean ± SD from three biological replicates. Statistical significance was determined using one-way ANOVA followed by Tukey’s multiple comparisons test. The combination treatment induced significantly higher ROS levels compared to control, polymyxin B, and vehicle alone (****P < 0.0001).

To determine whether hyperforin potentiates polymyxin B activity via oxidative stress, intracellular ROS levels were quantified using the DCFH-DA assay in *P. aeruginosa* FADDI-PA070 (Fig. 5). Polymyxin B treatment alone modestly increased ROS production (160 ± 8 RFU; 1.6-fold increase vs control), while hyperforin monotherapy produced a similar effect (145 ± 7 RFU; 1.45-fold increase). In contrast, the combination of polymyxin B and hyperforin resulted in a markedly higher ROS signal (280 ± 12 RFU; 2.8-fold increase), indicating a significant enhancement in oxidative stress compared to either agent alone (*P* < 0.0001, ANOVA with Tukey’s post hoc test).

These data are consistent with increased oxidative stress under combination treatment and, together with the membrane assays, support a complementary role for redox effects without assigning causality.

### Cytotoxicity and hemolysis assays

We evaluated hemolytic activity in human erythrocytes and cytotoxicity toward HEK-293 cells. Hyperforin showed no measurable hemolysis up to 640 µg/mL (HC₁₀ and HC₅₀ >640 µg/mL across three donors), whereas the positive control melittin lysed RBCs with HC_10_ = 5.76–6.21 µg/mL and HC_50_ = 11.3–13.5 µg/mL (Table S2). In the HEK-293 cytotoxicity assay, hyperforin did not reach 50% growth inhibition at 640 µg/mL in two independent experiments (CC_50_ > 640 µg/mL), while the control tamoxifen displayed CC_50_ values of 26.1 and 46.1 µg/mL (Table S3). Thus, no measurable hemolysis or HEK-293 cytotoxicity was detected up to 640 µg/mL, >160-fold higher than the 4 µg/mL hyperforin level used in the synergy assays, supporting a wide *in vitro* exposure margin.

## DISCUSSION

Hyperforin, though inactive on its own against Gram-negative pathogens, significantly potentiated the activity of polymyxin B in combination treatment regimens. This synergistic interaction was consistently observed across polymyxin-susceptible and polymyxin-resistant isolates of *P. aeruginosa*, *K. pneumoniae*, and *A. baumannii*, including highly resistant strains with polymyxin MICs up to 128 mg/L. Most notably, in *P. aeruginosa* FADDI-PA070, where polymyxin B alone was ineffective, the combination achieved >7-log₁₀ bacterial killing at 24  h.

Time-kill assays consistently showed enhanced and sustained bactericidal activity when polymyxin B was combined with hyperforin. In most cases, monotherapy led to regrowth by 24 h, whereas the combination maintained bacterial suppression. This was especially evident in resistant strains, highlighting the potential of this strategy to overcome clinically relevant polymyxin resistance (4, 5).

SEM and TEM revealed extensive membrane and intracellular disruption following combination treatment, consistent with a membrane-targeting effect. SEM showed pronounced surface deformation and collapse, while TEM demonstrated cytoplasmic disintegration and vacuole formation, features absent following treatment with either agent alone. While not mechanistic in isolation, these ultrastructural changes are consistent with membrane-targeted synergy between polymyxin B and hyperforin, contributing to enhanced bacterial killing (6, 7).

To further assess membrane-disruptive activity, we conducted an NPN uptake assay to evaluate OM permeability. Hyperforin significantly enhanced NPN uptake in combination with polymyxin B compared to either agent alone, indicating increased OM destabilization. This supports a mechanism in which hyperforin facilitates or amplifies polymyxin-induced membrane permeabilization, particularly in polymyxin-resistant strains. In addition to its membrane-disruptive effects, hyperforin also appears to enhance oxidative stress within bacterial cells. Our ROS assay results showed a significant increase in intracellular ROS generation following combination treatment, compared to either monotherapy. This finding suggests that the synergistic killing effect may involve both membrane perturbation and redox imbalance, a dual mechanism that has been observed with other polymyxin-based combinations (33–35). Oxidative damage can further compromise cellular integrity and overwhelm bacterial repair systems, particularly in resistant strains like *P. aeruginosa* FADDI-PA070. Taken together, these findings support a model in which polymyxin B and hyperforin act in a complementary manner. Polymyxin B binds to LPS and permeabilizes the OM, thereby facilitating the entry of hyperforin (6). Once inside, hyperforin’s lipophilic, protonophore-like properties likely exacerbate membrane destabilization (36), amplifying bacterial killing. Such multi-targeted stress provides a plausible explanation for the consistent synergy observed across both susceptible and resistant strains. These mechanistic readouts (SEM/TEM, NPN, and ROS) are correlative and were performed in a single polymyxin-resistant *P. aeruginosa* isolate; future work will test causality (e.g., ROS quenching and membrane-stabilizing conditions) and extend to additional MDR clinical isolates.

Interestingly, synergy was also observed in polymyxin-susceptible strains, suggesting the benefit of combination therapy may extend beyond resistant isolates. This broader application could be particularly valuable in reducing the risk of resistance emergence during monotherapy, a well-known limitation of polymyxins in clinical settings (37–39).

The LPS-deficient strain *A. baumannii* FADDI-AB065 was uniquely sensitive to hyperforin alone (MIC 0.25 mg/L), possibly due to its compromised OM (10, 40). This finding supports the idea that the target of hyperforin may lie beyond the OM and its effectiveness improves when membrane barriers are disrupted (6, 10).

Biofilm eradication data further reinforce the clinical potential of the combination. Polymyxin B and hyperforin together significantly reduced biofilm biomass at concentrations achievable via inhaled therapy (31, 32), a promising route for treating chronic respiratory infections caused by Gram-negative pathogens. It should be noted, however, that the extent of eradication observed in biofilm assays was less pronounced than the synergy seen in time-kill studies. This discrepancy reflects the fundamental difference in susceptibility between planktonic and biofilm-associated bacteria. Mature biofilms possess protective mechanisms such as restricted antibiotic penetration, altered metabolic activity, and the presence of persister subpopulations, all of which contribute to intensified tolerance. These features explain why concentrations effective against planktonic cells fail to achieve comparable eradication of established biofilms, consistent with well-documented challenges in biofilm therapy (41, 42).

Although the combination of polymyxin B and hyperforin demonstrated strong *in vitro* activity, the clinical applicability of hyperforin remains uncertain due to its poor systemic bioavailability and low plasma concentrations (~25–500 ng/mL) (13, 14, 43). In our biofilm eradication assays, the combination exceeded the prespecified 70% eradication threshold at 16 mg/L polymyxin B, a concentration well within the epithelial lining fluid (ELF) levels reported in a recent therapeutic drug monitoring study. Following aerosolized and intravenous administration of polymyxin B in ventilator-associated pneumonia patients, ELF concentrations ranged from 20.6 to 97.6  mg/L, while plasma levels remained between 1.19 and 5.16  mg/L. The ELF AUC_0–24_ values reached as high as 1,872.9  mg·h/L, providing robust support for achieving pharmacologically effective exposures at the site of infection through inhaled therapy. These findings support the feasibility of co-delivering polymyxin B and hyperforin via local targeted lung administration, such as dry powder inhalation or nebulized formulations as testable rationale, to maximize efficacy at the infection site while limiting systemic toxicity (31). In dedicated safety assays, hyperforin caused no measurable hemolysis up to 640 µg/mL (HC_10_/HC_50_ >640 µg/mL) and did not reach 50% cytotoxicity in HEK-293 cells up to 640 µg/mL (CC_50_ > 640 µg/mL), providing a >160-fold *in vitro* exposure margin relative to the 4 µg/mL used for synergy. This is consistent with prior *in vivo* toxicology of oral hyperforin ethylenediammonium salt showing no lethality at 2,000–5,000 mg/kg (p.o.; Class V, LD_50_ >5,000 mg/kg) and reversible elevations in AST/ALT on subacute dosing (44).

Overall, this study supports the potential of hyperforin as an adjuvant that potentiates polymyxin B against MDR Gram-negative pathogens. Future work will focus on causal mechanistic tests, expansion to additional MDR clinical isolates across species, formulation optimization for local delivery, and *in vivo* validation to assess translational feasibility.

## SUPPLEMENTAL MATERIAL

Supplemental material aac.01007-25-s0001.docx Fig. S1; Tables S1 to S3.

ASM does not own the copyrights to Supplemental Material that may be linked to, or accessed through, an article. The authors have granted ASM a non-exclusive, world-wide license to publish the Supplemental Material files. Please contact the corresponding author directly for reuse.
