# Supplementary material for: Hyperforin potentiates polymyxin B against multidrug-resistant Gram-negative pathogens via membrane disruption, biofilm eradication, and oxidative stress
Source: Antimicrob Agents Chemother. 2025 Nov 12;69(12):e01007-25. doi: 10.1128/aac.01007-25 (PMC12691686; doi:10.1128/aac.01007-25)
Supplement: Supplemental material — Fig. S1; Tables S1 to S3. [file aac.01007-25-s0001.docx]

**Supplementary Information: Hyperforin Potentiates Polymyxin B against Multidrug-Resistant Gram-Negative Pathogens via Membrane Disruption, Biofilm Eradication, and Oxidative Stress**

Maytham Hussein,^1*^ Simon Crawford,^2^ Mark Baker,^3^ Holly Floyd,^4^ Rafah Allobawi,^1^ Mark A. T. Blaskovich,^5^ Gauri G. Rao,^6^ Johannes Zuegg,^4^ Jian Li,^7^ Tony Velkov^1^*

**Affiliations:**^1^Monash Biomedicine Discovery Institute, Department of Pharmacology, Monash University, Clayton, Victoria, 3800, Australia. ^2^Monash Biomedicine Discovery Institute, Department of Microbiology, Monash University, Clayton, Victoria, 3800, Australia; ^3^Discipline of Biological Sciences, Priority Research Centre in Reproductive Biology, Faculty of Science and IT, University of Newcastle, University Drive, Callaghan, NSW, 2308, Australia; ^4^ Institute of Molecular Biosciences, The University of Queensland, Brisbane, QLD, 4072, Australia; ^5^Centre for Superbug Solutions and ARC Training Centre for Environmental and Agricultural Solutions to Antimicrobial Resistance, Institute for Molecular Bioscience, The University of Queensland, St Lucia, QLD, 4072, Australia;^6^Titus Family Department of Clinical Pharmacy, University of Southern California, 1985 Zonal Avenue, Los Angeles, CA 90089-9121, USA; ^7^Monash Biomedicine Discovery Institute, Department of Microbiology, Monash University, Clayton, Victoria, 3800, Australia

*Corresponding author: tony.velkov@monash.edu; maytham.hussein.old@monash.edu

**Keywords.** Polymyxin B, Hyperforin, Antimicrobial Resistance, Synergy, Biofilm eradication.

**Short Title:** Hyperforin-Polymyxin B Synergy in MDR Gram-Negatives

**Figure S1.** Time-kill kinetics of polymyxin B (PMB), hyperforin (HF), and their combination against clinical strains of *P. aeruginosa*, *A. baumannii*, and *K. pneumoniae*. PMB-susceptible strains: *A. baumannii* ATCC 17978 (MIC 1 mg/L), *K. pneumoniae* FADDI-KP003 (MIC 1 mg/L). PMB-resistant strains: *P. aeruginosa* FADDI-PA006 (MIC 8 mg/L), *P. aeruginosa* FADDI-PA064 (MIC ≥128 mg/L), *A. baumannii* FADDI-AB225 (32 mg/L), *K. pneumoniae* FADDI-KP003 (32 mg/L). HF MICs were >128 mg/L for all strains. Data are presented as means of three independent cultures. Vertical bars represent standard deviations; error bars are too small to be visible in the graphs.

**
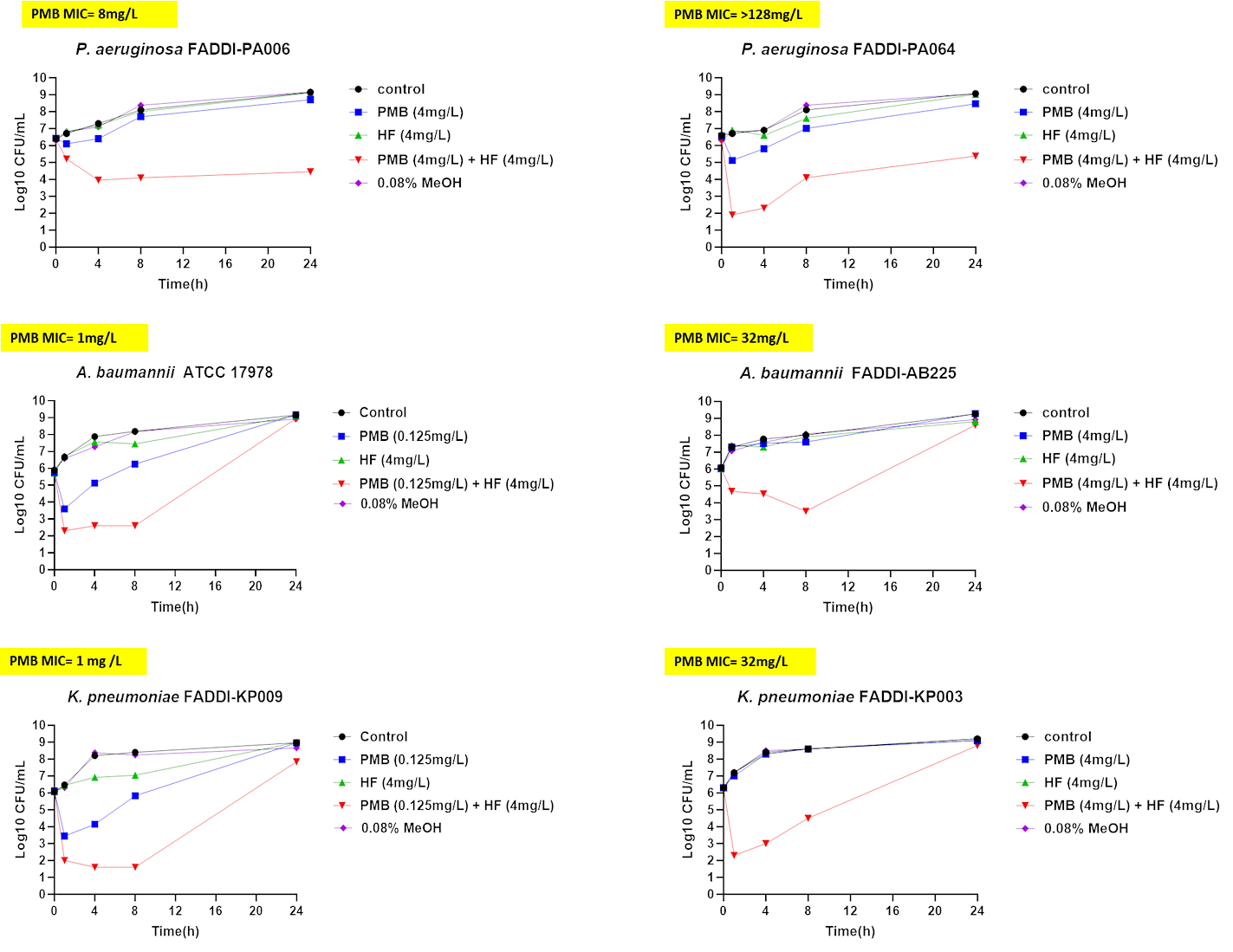
**

**Supplementary Table S1.** Antimicrobial susceptibility profile of *Pseudomonas aeruginosa* strain FADDI-PA067 by disk diffusion. Interpretations followed the Clinical and Laboratory Standards Institute guideline^1^.

| Antibiotic | S/I/R |
| --- | --- |
| Aztreonam | S |
| Ceftazidime | R |
| Ciprofloxacin | S |
| Gentamicin | R |
| Meropenem | R |
| Piperacillin | S |
| Ticarcillin | R |
| Tobramycin | R |

S = susceptible; I = intermediate; R = resistant; ND = not determined.

**Supplementary Table S2.** Hemolysis of human erythrocytes (from whole blood) by hyperforin and melittin (positive control). HC_10_ and HC_50_ (µg/mL) are reported for *n* = 3 donors (independent experiments).

| **Compound name** | **HC_10_ (µg/mL)** | **HC_50_ (µg/mL)** |
| --- | --- | --- |
| Melittin | 5.76 | 11.64 |
|  | 6.21 | 13.52 |
|  | 5.76 | 13.3 |
| Hyperforin | >640 | >640 |
|  | >640 | >640 |
|  | >640 | >640 |

*->640 indicates that the effect did not reach 10% or 50% at the highest tested concentration (640 µg/mL).*

*-HC_10_/HC_50_ = concentrations causing 10% and 50% hemolysis.*

**Supplementary Table S3.** Cytotoxicity against HEK-293 cells (ATCC CRL-1573). CC₅₀ (µg/mL) values from four-parameter logistic fits for two independent experiments (each concentration in ≥3 technical replicate wells; technical replicates averaged prior to fitting).

| **Compound Name** | **Exp 1 CC₅₀ (µg/mL)** | **Exp 2 CC₅₀ (µg/mL)** |
| --- | --- | --- |
| Tamoxifen (Control) | 26.06 | 46.13 |
| Hyperforin | >640 | >640 |

*->640 indicates CC₅₀ was not reached at the highest concentration tested (640 µg/mL).*

*-CC₅₀ = concentration that reduces cell viability by 50% relative to the vehicle control (estimated from a four-parameter logistic fit).*

**Reference**

(1) Clinical and Laboratory Standards Institute. *Performance Standards for Antimicrobial Susceptibility Testing. 34th ed. CLSI Supplement M100.*; Wayne, PA, 2024.
